# Text and Structural Data Mining of Influenza Mentions in Web and Social Media

**DOI:** 10.3390/ijerph7020596

**Published:** 2010-02-22

**Authors:** Courtney D. Corley, Diane J. Cook, Armin R. Mikler, Karan P. Singh

**Affiliations:** 1 Pacific Northwest National Laboratory, 902 Battelle Blvd., Richland, WA 99352, USA; 2 School of Electrical Engineering and Computer Science, Washington State University, PO Box 642752 Pullman, Washington 99164, USA; E-Mail: cook@eecs.wsu.edu; 3 Department of Computer Science and Engineering, University of North Texas, 1155 Union Circle #311366 Denton, TX 76203, USA; E-Mail: mikler@unt.edu; 4 Department of Biostatistics, University of North Texas Health Science Center, 3500 Camp Bowie Blvd. Fort Worth, TX 76107, USA; E-Mail: ksingh@hsc.unt.edu

**Keywords:** disease surveillance, public health epidemiology, health informatics, graph-based data mining, web and social media, social network analysis

## Abstract

Text and structural data mining of web and social media (WSM) provides a novel disease surveillance resource and can identify online communities for targeted public health communications (PHC) to assure wide dissemination of pertinent information. WSM that mention influenza are harvested over a 24-week period, 5 October 2008 to 21 March 2009. Link analysis reveals communities for targeted PHC. Text mining is shown to identify trends in flu posts that correlate to real-world influenza-like illness patient report data. We also bring to bear a graph-based data mining technique to detect anomalies among flu blogs connected by publisher type, links, and user-tags.

## 1. Introduction

Influenza diagnosis based solely on the presentation of symptoms is limited as these symptoms may be associated with many other diseases. Serologic and antigen tests require that a patient with influenza-like illness (ILI) be examined by a physician who can either conduct a rapid diagnostic test or take blood samples in a laboratory testing. This suggests that many cases of influenza remain undiagnosed. While the presence of influenza in an individual can be confirmed through specific diagnostic tests, the influenza prevalence in the population at any given time is unknown and can only be estimated. In the past, such estimates have relied solely on the extrapolation of diagnosed cases, making it difficult to identify the various phases of seasonal influenza or to identify a more serious manifestation of a flu epidemic.

**Figure 1.**
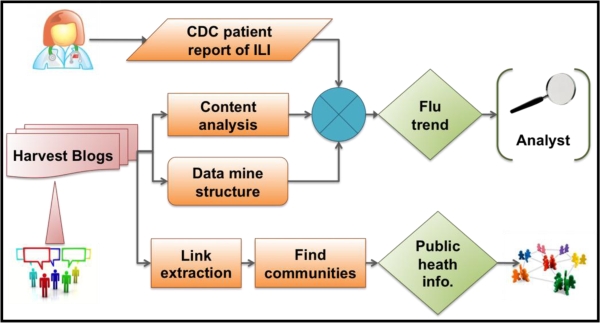
Methodology to monitor influenza-like illness in social media and to identify possible web and social media communities to participate in a public health response.

Web and social media (WSM) provide a resource to detect increases in ILI. This paper evaluates blog posts, a type of WSM, that discuss influenza and the analyses show a significant correlation with patient reporting of ILI during the US 2008–2009 influenza season. Preliminary experimental results on data covering two months in 2008 have been published in conference proceedings [1]. In this article, we present comprehensive analysis, covering 24 months of data. A well-defined response strategy to an outbreak may make use of WSM to reduce population and human impact of the disease. We suggest a possible response that identifies WSM influenza-related communities that share flu-related postings. These community or crowd sources could broker and disseminate important intervention information in the case of an infectious disease outbreak. Our proposed framework, in Figure 1, visually describes this approach to detecting and responding to influenza epidemics.

We briefly discuss a history of infectious disease outbreaks and recent approaches in online public health surveillance of influenza. We also discuss the value of social community with regard to outbreak responses. Next, the data set used in our analysis is presented and the methodology for information extraction and trend analysis is outlined. Through discovery and verification of trends in influenza-related blogs, we verify a correlation to Centers for Disease Control and Prevention (CDC) ILI patient reporting at sentinel healthcare providers. Additionally, categories, frequency, and influenza-post persistence qualitatively assist ILI trend identification in blogs. Strongly connected communities are evaluated and influential bloggers identified that should be part of a WSM outbreak response. Then we leverage graph-based data mining to further identify structural anomalies in the flu blogosphere that correspond to increases in ILI.

### Using Web and Social Media for Biosurveillance

The pervasiveness and ubiquity of internet resources provide individuals with access to many information sources that facilitate self-diagnosis and provide means for nontraditional biosurveillance; for example, one can combine specific disease symptoms to form search queries. The results of such search queries often lead to sites that may help diagnose the illness and offer medical advice (e.g., PeopleLikeMe.com, WebMD.com). Recently, Google™ has addressed this issue by capturing the query keywords and identifying specific searches involving search terms that indicate ILI [2]. Published research on influenza internet surveillance also includes search “advertisement click-through” [3], using a set of Yahoo search queries containing the words “flu” or “influenza” [4], and health website access logs [5,6]. Other information sources, such as telephone triage services, can be useful for ILI detection. The findings in Yih *et al*. [7] show that telephone triage service is not a reliable measure for influenza surveillance due to service coverage; however, it may be beneficial in certain situations where other surveillance measures are inadequate. We envision several applications that leverage automatic open source document analytics for biosurveillance: such a system could provide lagging indicators of a disease outbreak to a component of a US port and border’s biosurveillance system (Personal communication with Dr. Andrew Plummer, Centers for Disease Control and Prevention, National Center for Preparedness, Detection, and Control of Infectious Diseases, Division of Global Migration and Quarantine); a second application in development is the recently EU-funded project Medical Ecosystem Personalized Event-Based, and a hypothetical third application could provide workflow in existing global surveillance systems (such as Argus Global at Georgetown University) that must employ linguists to curate bio-event notices.

## 2. Data and Methods

### 2.1. Data

**Figure 2.**
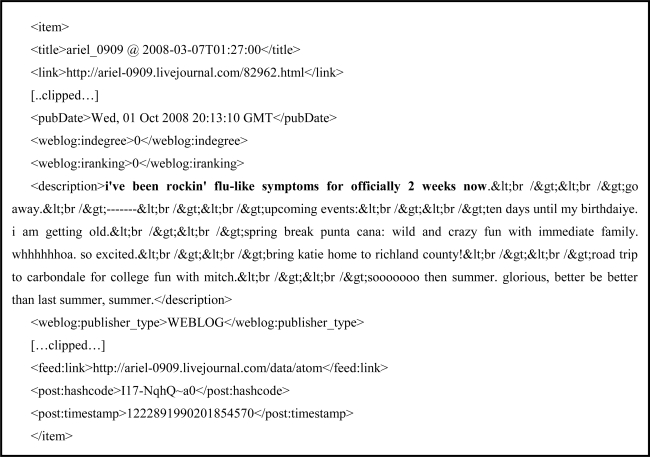
Example XML encoding of social media post that mentions *flu*.

Spinn3r [8] is a WSM indexing service that conducts real-time indexing of all blogs, with a throughput of over 100,000 new blogs indexed per hour. Blog posts are accessed through an open source Java application programming interface (API). Metadata available with this data set (see Figure 2. Example XML encoding of social media post that mentions flu.) includes the following (if reported by source): blog title, blog URL, post title, post URL, date posted (accurate to seconds), description, full HTML encoded content, subject tags annotated by author, and language.

**Figure 3.**
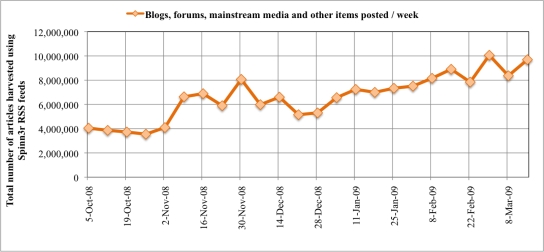
Blogs, forums, mainstream media, and other articles harvested using Spinn3r RSS/ATOM feeds, 5 October 2008 to 21 March 2009.

**Figure 4.**
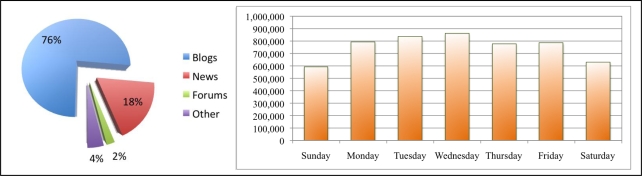
(a) Web and social media publisher types (%) from 158,497,700 items posted over 24 weeks. (b) Per day of week blogs, forums, mainstream media and other items averaged over 24 weeks, 5 October 2008 to 21 March 2009.

Data are selected from an arbitrary time period of 24 weeks, beginning 5 October and ending 21 March 2009. A total of 158,497,700 WSM items were pulled from Spinn3r RSS and ATOM feeds. We identified a significant increase in blog coverage resulting from the success of Spinn3r service and subsequent expansion of web crawlers in addition to organic growth of WSM publishing, as shown in Figure 3. It is evident from the average number of blogs posted per day of week summarized in Figure 4b that most WSM in this data are published during the week and less so on the weekends. A majority of the articles we analyzed were weblogs (labeled by Spinn3r); mainstream media accounts for 20% of the data and the remaining types include forums and classified ads (see Figure 4a). In the analysis reported here, we select English language WSM items indexed by Spinn3r when a lexical match exists to the terms *influenza* and *flu* anywhere in its content (misspellings and synonyms are not considered). The blog items are grouped by month, week (Sunday to Saturday), and day of week. The extracted blog items containing influenza keywords are herein termed flu-content posts or **FC-posts**. Missing from our data are more recent evolutions of WSM such as micro-blogs, wikis, and deep-web communities that many times are gated and not indexed in shallow web crawls.

Indexing, parsing, and link extraction code was written in Python, parallelized using pyMPI and openmpi and executed on an eight-node cluster (2.66 GHz Quad Core Xeon processors), with 64 core, 256 GB memory, 30 TB of network storage [9,10]. This compute resource is housed at the University of North Texas Center for Computational Epidemiology and Response Analysis.

### 2.2. Analysis

#### 2.2.1. Text mining to monitor influenza trends

Text mining is the process of discovering information in large text collections and automatically identifying interesting patterns and relationships in textual data [11]. Text mining is particularly related to data mining, an older research area focused on the extraction of significant information from data records. However, text mining has proven to be more difficult than data mining, as the source data consists of unstructured collections of documents rather than structured databases. A large number of applications now utilize text mining, including question-answering applications, automatic construction of databases on job postings, and dictionary construction. Feldman and Sanger [12] have recently published a thorough survey of research work in the area of text mining.

Influenza WSM item trends can be monitored using the social media mining methodology presented in this paper. This methodology facilitates identification of outbreaks and increases of influenza infection in the population. We posit a strong correlation exists between the frequency of FC-posts per week and CDC ILI surveillance data. Qualitative assessment of category tags, prevalence of FC-posts on a blog site, and persistent posting of flu-related posts also suggest ILI trends.

We hypothesize that the frequency of blog-world flu posts correlate with a patient reporting ILI and the US flu season. To verify this hypothesis, we compare our data to CDC surveillance reports from sentinel healthcare providers. The CDC website states the Outpatient Influenza-like-illness Surveillance Network (ILINet) consists of about 2,400 healthcare providers in 50 states reporting approximately 16 million patient visits each year. Each provider reports data to CDC on the total number of patients seen and the number of those patients with ILI by age group. For this system, ILI is defined as fever (temperature of 100 °F [37.8 °C] or greater) and a cough and/or a sore throat in the absence of a known cause other than influenza [13].

#### 2.2.2. Graph-based structure mining to discover blog flu communities and anomaly detection

WSM communities will play a vital role in any public health response to an outbreak. Influential bloggers can disseminate and broker response strategies and interventions in their blog communities. These bloggers could be first responders to a disease outbreak, in an information sense. Their readers will hopefully trigger an information cascade, spreading public health communications (*i.e.*, to vaccinate, quarantine, close schools, *etc*.). Although considerably less costly than a mainstream media campaign, a WSM targeted response must be cost-effective and optimized to achieve maximum strategy penetration. Any blogger participating in a public health campaign needs to have influence in their community and the ability to disseminate information to other WSM. Closeness and betweenness centrality measures and Google’s PageRank (eigenvector centrality) will rank influenza community blog sites in order to target key actors. Additionally, the Girvan-Newman community finding algorithm will identify communities of interest.

Moreover, graph-based algorithms can be leveraged not only to identify communities but also to facilitate bio-event detection by searching for anomalies in the link-structure of WSM. Numerous approaches have been developed for discovering concepts in linear, attribute-value databases. Current data mining research focuses primarily on algorithms to discover sets of attributes that can discriminate data entities into classes, such as shopping or banking trends for a particular demographic group. These approaches are difficult when key concepts involve relationships between the data points. In contrast, we are developing data mining techniques to discover patterns consisting of complex relationships between entities. We have introduced a method for discovering substructures in structural databases implemented in the Subdue system [14]. In contrast with alternative approaches, Subdue is devised for general-purpose automated discovery, concept learning, and hierarchical clustering. Hence, the method can be applied to many structural domains. Subdue is leveraged in our analysis to identify non-obvious patterns in blog posts that may serve as lagging-indicators of an influenza outbreak.

#### 2.2.3. Potential problems and associated risks

There are associated risks with using open source documents obtained through WSM primarily due to sample bias because of limited access to technology and truthfulness of blogger statements. People that can afford home access to computers and the internet are usually educated and literate [15,16]. However, the ubiquity of wireless internet access in public places such as libraries, restaurants, and cafes enables users from a variety of social and educational levels to engage with and contribute to WSM. Second, the validity of self-reported health diagnoses or behaviors such as voluntary quarantine (staying home when sick), vaccination, and increased hygiene is unknown. This situation raises concerns and uncertainty as to whether these self-disclosures reflect intended, false, or actual behavior (diagnoses). Previous studies of self-reported behaviors via the internet have shown that computer use encourages high levels of self-disclosure and uninhibited personal expression. This finding supports the validity of internet self-reporting; however, formal study is needed to verify the accuracy of self-reported diagnoses and behaviors in WSM.

## 3. Results and Discussion

The CDC ILINet surveillance and FC-post per week data are plotted in. CDC ILI symptoms per visit at sentinel US healthcare providers label the primary Y-axis. The secondary Y-axis marks the FC-post per week frequency normalized by the total number of posts in the 24-week period. Correlation between the two data series is measured with a Pearson correlation coefficient, r. To prove our hypothesis that a correlation exists between CDC ILINet reports and mined WSM FC-post frequency, Pearson's correlation statistic is evaluated between the two data series. The Pearson statistic evaluates to unity if the two data series are exactly matching, r = 1. If no correlation exists between the data series, the Pearson statistic evaluates to zero, r = 0. In our analysis, the 24 ILI and FC-post data points correlate strongly with a high Pearson statistic, r = 0.545, and the correlation is significant with 95% confidence. Notice the deviation in the time series at 1 February to 21 March 2009. After close inspection of the data provided by Spinn3r, we identified a significant increase in blog coverage resulting from the success of their service and subsequent expansion of web crawlers, thereby biasing the influenza blog presence normalization. Moreover, graph-base data mining discovered substantial presence of MySpace blogs in the last three weeks of data. We manually inspected the blogs and discovered many of the MySpace blogs were discussing the health of American Idol contestants, several of whom were sick with the flu.

**Figure 6.**
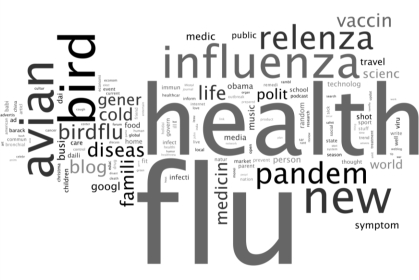
Wordle of most frequent author-tagged categories (stemmed). [influenza posts: 5 October 2008 to 21 March 2009].

Each WSM item has rich metadata that can be leveraged for content analysis. A folksonomy is defined from WSM by associated author “tags” extracted from category metadata. Moreover, a folksonomy is a type of classification system for online content, created by an individual user who tags information with freely chosen keywords. The Porter stemming algorithm [17] is used to find the morphological root from the author-tagged labels. Duplicate author-tagged labels are only counted once per blogger. Table 1 lists the top 45 categories and how often they appear. Figure 6 is a tag-cloud graphic, called a *Wordle* (see www.wordle.net), that visually depicts the frequency of categories in the data. The top categories (e.g., flu, health, bird, avian, influenza) are intuitive; however, one could monitor categories that imply self or close-proxy infection such as family, sick, symptom, home, school, and other representative terms.

**Figure 7.**
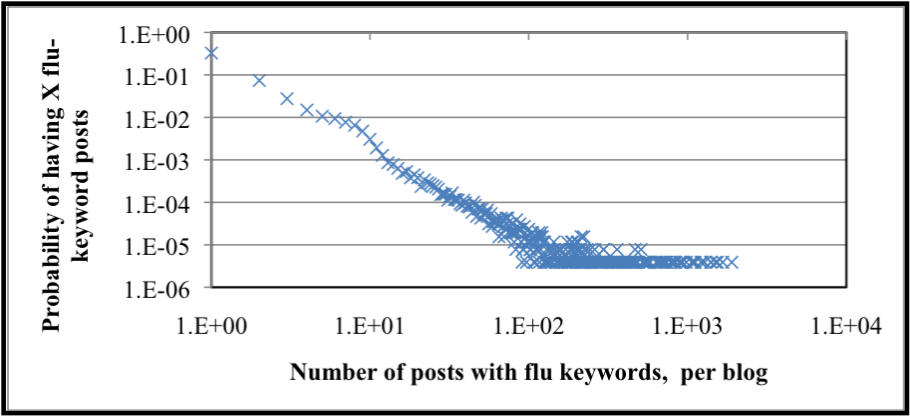
Cumulative probability distribution of the number of influenza posts, per blogger, 5 October 2008 to 21 March 2009.

Monitoring self-identification and secondhand FC-post trends can mark increases in ILI. It can be said that bloggers that post often about influenza are more likely to a) be an authority on influenza (perhaps not an expert, however) where its readers find information on influenza or b) the blogger is frequently sick with influenza. How often or how persistently bloggers author FC-posts indicates trends as well; a blog-site that has FC-posts for a limited time is more likely to be a first- or secondhand identification of ILI. The cumulative probability distribution for how many posts a blogger writes about influenza is graphically summarized in Figure 7. The number of posts with influenza keywords per blogger is plotted on the X-axis and the associated probability of a blogger posting X number of flu posts labels the Y-axis; the data is plotted on a log-log scale. We see from that over 95% of bloggers only post one “flu” post, whereas the most frequent flu post blogger authored 1,897 posts. The probability of a blogger posting 1,897 flu posts is approximately 0.0000388%. The heavy-tailed distribution is indicative of social processes (e.g., number of intimate partners [18]) and is present in the distribution of flu posts per blogger. The cumulative probability distribution also supports the hypothesis that most bloggers do not frequently author influenza content. Content analysis supporting the claim that less frequent posters self-identify ILI is left for future work.

To study the link-based structure of bloggers authoring influenza-related content, we extract the URLs linked in each blog post. These URLs and blog permalinks (829,662 URLs) are truncated to the network location and path resulting in 694,388 unique URLs. A link graph is then constructed from the blogger source URL and out-links from the influenza posts, removing self-references and parallel out-links and the largest weak component producing an aggregate graph of 694,388 nodes (bloggers) and 3,529,362 directed edges (unique blogger to blogger links).

Table 2 lists the seven most prolific flu bloggers and their degree (In, Out, and Total). The relatively low In degree supports the claim that frequent posters are news- and opinion-oriented and not always the most influential in online communities. Centrality metrics are evaluated on the same most frequent bloggers. The results are listed in Table 3. Three of the top posters have no in-links, implying they are spam blogs and have no influence in the “flu” blogosphere. We verify this statement with a quick hand-check of the URL. The RSS feed BirdFluMonitor has the highest out closeness centrality, but no In degree, implying they are adept at publishing links to popular blogs but are not influential themselves. Three blogs (h5n1, a flu diary, fluwikie2) are interesting hubs of the flu blogosphere. The blog “A Flu Diary” has the largest betweenness centrality (interpersonal influence) with high In and Out degree and demonstrates the capability to broker influential information in the target blogosphere. The blog h5n1 has the greatest PageRank and in-closeness centrality; moreover, it has the most published items of the most frequent bloggers and is influential in disseminating h5n1 information.

Using the most frequent flu bloggers is a naïve approach to finding target WSM communities to be leveraged for public health response. To advance our approach, we target strongly connected components within our flu link graph community identification (Flake, Lawrence, and Gilles definition of community [19,20]). The link graph’s largest strongly connected component is over 17,000 unique URLs. However, its nodes are spam bloggers; specifically, they were all LiveJournal blogs, and each post had exactly eight out-links The uniformity and a manual inspection of these as spam blogs show they were written for the purpose of search engine optimization. Therefore, we cluster the second largest strongly connect component, which consists of 2,306 blogs, 26,768 edges, and an average degree of 23. The Girvan-Newman community finding algorithm (recursively removes the node with the highest betweenness centrality) identifies 11 communities.

Table 4 reports centralities and size for the six largest communities. An interesting finding is that these communities are clustered not only by publisher types but also by parent company. Not surprisingly, the largest community comprises personal blogs and general reporting newspapers; the remaining consist of mainstream and local news outlets, international audience media, LiveJournal, and entertainment industry (e.g., Viacom, Reed), large news conglomerates (e.g., News Corp, Disney), and commentary, opinion and editorial content. A successful WSM public health campaign should have a presence and influence in each of the reported blog communities to ensure wide coverage and dissemination of pertinent information.

Detecting anomalies in various data sets is an important endeavor. We define an anomaly as a surprising or unusual occurrence. Using statistical approaches has led to various successes such as detecting computer and network intrusions. Recent research in graph-based anomaly detection has paved the way for new approaches that not only complement the non-graph-methods but also provide mechanisms for handling data that cannot be easily analyzed with traditional statistical approaches [21]. Again, Subdue can be used to address this challenge. Subdue examines an entire graph and reports unusual substructures, or substructures that occur infrequently, within it [14]. Subdue also takes into account the regularity of the data to determine how likely it is for a substructure to occur given the predictability of the structural data surrounding the substructure. These ideas have been tested in applications including intrusion detection and terrorist activity analysis.

**Figure 8.**
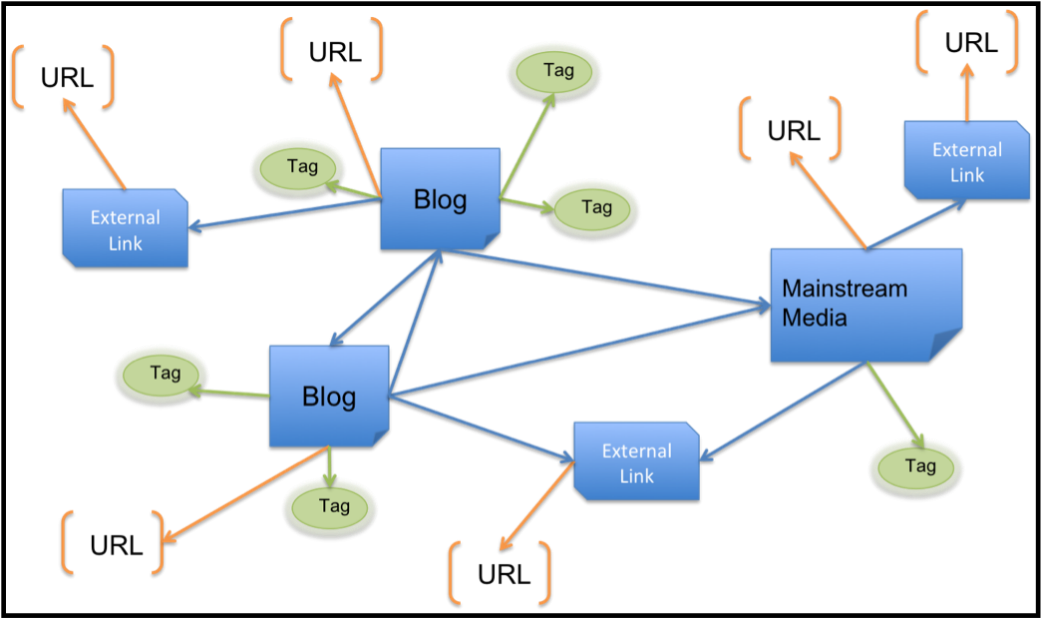
Example graph representation of influenza bloggers used for anomaly detection by Subdue.

To facilitate identification of ILI through graph-based data mining of influenza blogs, we base the representation on the link graph. Multiple posts by the same author are aggregated, representing a unique blogger; similarly, multiple tags and out and in links are only counted once per blogger. To structurally enrich the link graph, we connect the blogger URL and tags to a node labeled by the publisher type (e.g., blog, forum, mainstream media, external link) as depicted in Figure 8 Graph structures are created from weekly influenza blog posts to facilitate anomaly detection and correlation to CDC ILI patient reports. Figure 8 demonstrates how URLs are disaggregated from their WSM article, thereby creating a relationship between two entities (the WSM article and the URL). This allows Subdue to find informative subgraphs of blogs with differing content (news, personal blogs) in addition to traditional URL structures. The structurally enriched data and temporal format facilitate anomaly detection by Subdue (this is in contrast to the 24-week aggregate link graph used for community identification).

Table 5 lists the substructure features discovered by Subdue and identifies if they correspond to an anomaly for the purpose of outbreak detection. An analyst can then review the reported substructures for outbreak information. The first discovery of interest is during the week beginning 7 December 2008 identifying the UK Yahoo Answers site. During the same time frame, the United Kingdom was in the middle of its worst flu season in eight years. While correlating influenza post frequency to CDC ILINet data was unsuccessful in February and March 2009, Subdue is able to identify novel substructures in personal blogs that mention influenza. The third anomaly discovered by Subdue shows a high number of substructures occurrences, composed of MySpace blog posts discussing several American Idol contestants that contracted influenza and were unable to perform at their best during the weekly performance competition.

## 4. Methods and Materials

Wasserman and Faust state closeness can be productive in communicating information to other actors. It is defined in Equation 1 as the average shortest paths or geodesics distance from actor *v* and all reachable actors (*t* in *V*\*v*) in [22]:

(1) $$C_{C_{v}}=\frac{\sum_{t\in V\backslash v}d_{G}(v,t)}{n-1}$$

Betweenness centrality (Eqn. 2) measures interpersonal influence. Specifically, a blog is central if it lies between other blogs on their geodesics—the blog is “between” many others, where *g*_jk_ is the number of geodesics linking blog *j* and blog *k* [22] :

(2) $$C_{B_{v}}=\sum_{j<k}\frac{g_{jk}(n_{i}v,t)}{g_{jk}}$$

Page Rank is an example of eigenvector centrality and measures the importance of a **node** by assuming links from more central nodes contribute more to its ranking than less central nodes [23]. Let *d* be a damping factor (usually 0.85), *n* be the index to the node of interest, *p_n_* be the node, M(*p*_i_) be the set of nodes linking to *p*_n_ and L(*p*_j_) be the out-link counts on page *p*_j_:

(3) $$R_{p_{n}}=\frac{1-d}{N}+d\sum_{p_{j}\in M(p_{n})}\frac{PR(p_{j})}{L(p_{j})}$$

We take an intuitive and simple definition of WSM community and identify possible first responder bloggers by link analysis. Blog ranking enhances the idea that these communities can disseminate information as part of a broader public health response triggered by anomalies in ILINet and WSM surveillance. Community herein is defined similar to Flake, Lawrence, and Giles where there are more edges between member nodes than edges to external nodes. Formally, a community is a vertex subset *C in V*, such that for all vertices *v* ∈ *C*, *v* has at least as many edges connecting to vertices in *C* as it does to vertices in (V-C) [19,20]. Links from a non FC-post to an FC-post and vice versa are not defined in this community definition. The Girvan-Newman algorithm is used to identify communities in our data. The general form of this community structure finding algorithm is enumerated below, components remaining in the graph at the end of each iteration are the communities [24]:

1. Calculate betweenness scores for all edges in the network.
2. Find the edge with the highest score and remove it from the network. If two or more edges tie for highest score, choose one of them at random and remove that edge.
3. Recalculate betweenness for all remaining edges.
4. Repeat from step 2 until the desired number (if known a priori) of communities is reached, otherwise repeat from step 2 until no edges remain.

Subdue accepts as input directed or undirected graphs with labeled vertices (nodes) and edges (links), and outputs graphs representing the discovered pattern or learned concept. Formally, Subdue uses a labeled graph G = (V,E,L) as both input and output, where V = {v_1_, v_2_, …, v_n_} is a set of vertices, E = {(v_i_, v_j_) | v_i_, v_j_ ∈ V} is a set of edges, and L is a set of labels that can appear on vertices and edges. The graph G can contain directed edges, undirected edges, self-edges, and multi-edges. As an unsupervised algorithm, Subdue searches for a substructure, or subgraph of the input graph, that best compresses the input graph. Subdue uses a variant of beam search for its main search algorithm. A substructure in Subdue consists of a subgraph definition and all its occurrences throughout the graph.

**Figure 9.**
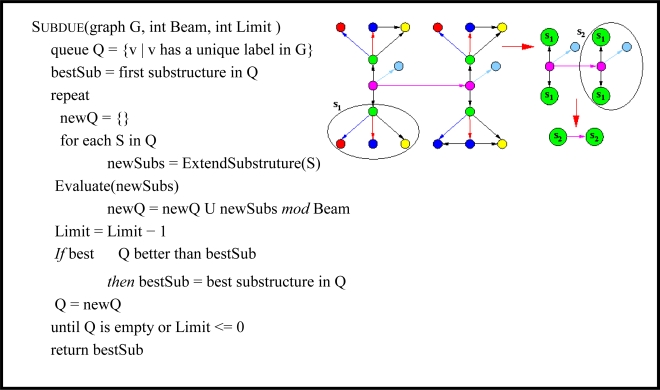
Subdue’s discovery algorithm and an example. The figure shows the discovered pattern (S_1_) from the original graph, the substructure found during the second iteration (S_2_), and the final graph compressed using substructures S_1_ and S_2_.

Subdue uses a polynomial-time beam search for its discovery algorithm, as summarized in Figure 9. The initial state of the search is the set of substructures consisting of all uniquely labeled vertices. Search progresses by applying the ExtendSubstructure operator to each substructure in the current state. As its name suggests, it extends a substructure in all possible ways by a single edge and a vertex or by only a single edge if both vertices are already in the subgraph. The resulting new substructures are ordered based on their compression (sometimes referred to as *value*) as calculated using the Minimum Description Length (MDL) [21] principle described below, and the top substructures (as determined by the beam) remain on the queue for further expansion.

Search terminates upon reaching a limit on the number of substructures extended or upon exhaustion of the search space. Once the search terminates and Subdue returns the list of best substructures, the graph can be compressed using the best substructure. The compression procedure replaces all instances of the substructure in the input graph by single vertices, which represent the substructure definition. Incoming and outgoing edges to and from the replaced instances will point to or originate from the new vertex that represents the instance. The Subdue algorithm can be invoked again on this compressed graph. As an example Figure 9 shows patterns that Subdue discovers in an example input graph and a compressed version of the graph.

## 5. Future Work

Emerging infectious diseases continue to have an impact on the health, safety, and sustainable growth of our nation as shown by the 2009 novel influenza A/H1N1 strain. Upon initial identification of widespread H1N1 outbreaks in April 2009, the CDC participated in a global concerted effort to control transmission of influenza A/H1N1 and prevent pandemic outbreaks by issuing public health response recommendations. Future work will quantify the impact and validate the use of WSM to monitor seasonal influenza epidemics and global pandemics. Preliminary influenza blog harvests during this pandemic, not including mentions on micro-blogging platforms (Twitter), are reported in Table 6. Geo-location tagging is now implemented in blog, social network, and micro-blogging platforms and future research will leverage this new data in the next-generation WSM biosurveillance system; however, geo-location information was not available in analyses reported here. Research is continuing on both health blogs and health micro-blogs to inform a robust disease surveillance system using open source documents.

**Figure 10.**
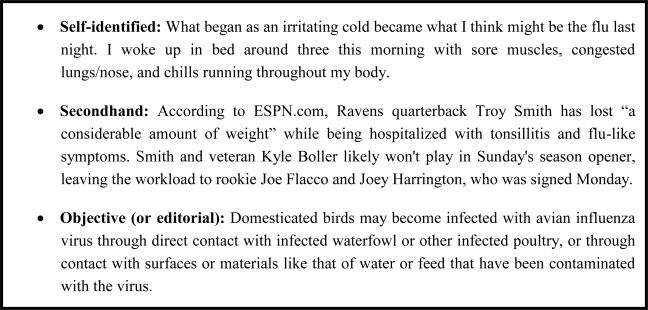
Three perspective blog posts that mention influenza: self-identification, secondhand, and objective/editorial.

Once influenza WSM items have been extracted, one can further monitor influenza outbreaks by evaluating the perspective of blog authors. Bloggers with a direct knowledge of influenza infection are more valuable to disease surveillance than those who author objective or opinion items. Identifying the perspective of influenza keyword posts facilitates determining its contribution to disease surveillance. Three author perspectives are identified in Figure 10. An FC-post can be (1) a self-identification of having ILI symptoms, (2) a secondhand (or by proxy) post about another individual having ILI, or (3) an opinion or objective article containing ILI keywords. Secondhand knowledge can be writing about a friend, schoolmate, family-member, or co-worker, but a blogger could also post details on a famous individual such as an athlete. The season opening of American football coincides with the data, and many FC-posts identify athletes who are unable to play because of an ILI. Automatic classification of the influenza post author’s perspective is ongoing research.

## 6. Conclusions

Text and structural data mining of WSM provides a novel disease surveillance resource and technique to identify online “flu” topic health information communities. Our proposed framework of complementary data-mining methods supports our hypothesis. We comprehensively evaluate blog posts containing influenza topic keywords through text, link, and structural data mining. Results from analysis show strong co-occurrence of flu blog posts during the US 2008–2009 flu season. That is, from 5 October 2008 to 21 March 2009, a high correlation exists between the frequency of posts, containing influenza keywords, per week and CDC ILI surveillance data. Frequency of flu posts per blogger follows a heavy-tailed distribution, and we show through graph metrics that the most prolific bloggers are not the most influential. Pertinent health information should have a presence in all identified WSM communities. The Girvan-Newman algorithm is leveraged to identify clusters of similar sites as potential target communities for online health information campaigns. The results show distinct WSM communities clustered by publisher and content type, such as News Corp & Disney properties, international audiences, or personal blogs.

Harvesting WSM is a continuing challenge with the explosive growth of internet usage. To complement the text mining approach to ILI monitoring, we apply a graph-based data mining technique, Subdue, to detect anomalies and informative substructures among flu blogs connected by publisher type, links, and user-tags. This technique flags anomalies not discovered with content analysis that correspond to the United Kingdom’s worst influenza season in eight years and the emergence of strong personal blog communications during the U.S. seasonal influenza peak incidence.

Link analysis reveals communities, clustered by content and in many cases corporate ownership, which should be targeted in a successful public health communications campaign to assure wide dissemination of pertinent information. Text mining of influenza mentions in WSM is shown to identify trends in flu posts that correlate to real-world ILI patient reporting data. Moreover, graph-based data mining is able to identify significant anomalies in flu blogs that were not identified through text analysis and can be flagged for further investigation by an analyst.

## Figures and Tables

**Figure 5.**
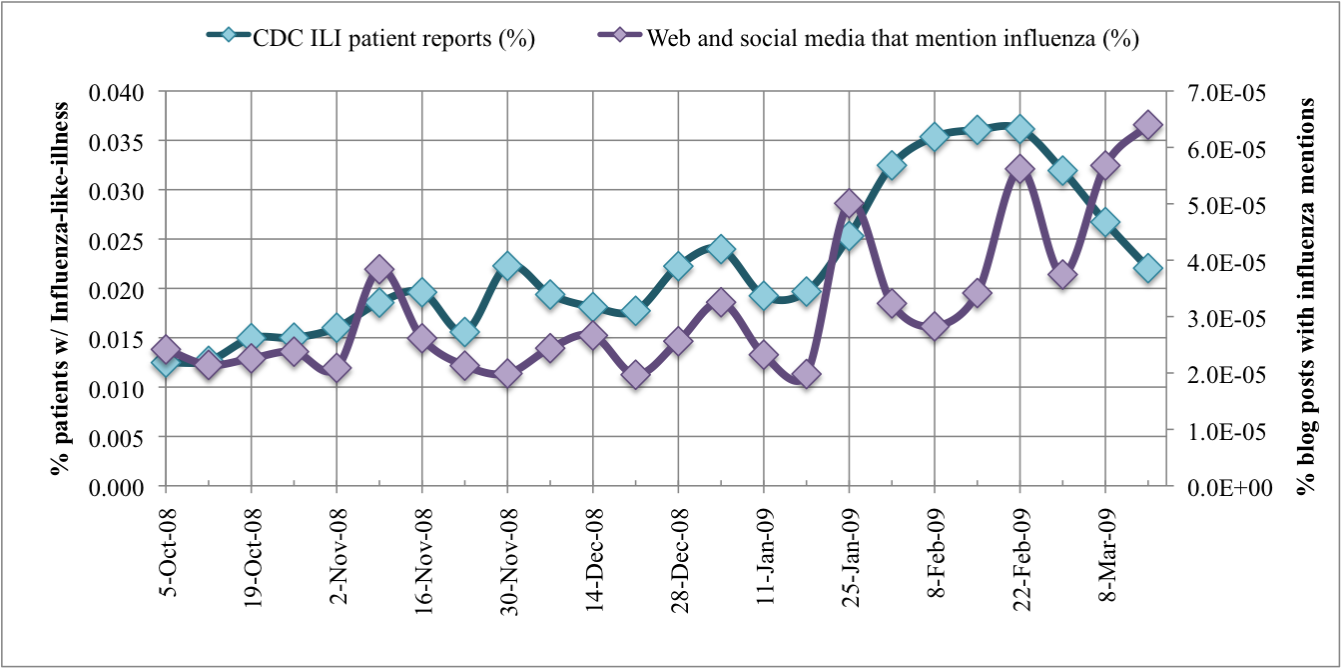
CDC ILINet *vs.* normalized blog post (with flu keywords) frequency per week. 5 October 2008 to 21 March 2009.

**Table 1.** Most frequent author-tagged categories (stemmed) [influenza posts: 5 October 2008 to 21 March 2009].

Flu	5605	medicin	697	shot	306
health	3946	gener	652	dai	297
bird	2030	polit	591	food	293
avian	1968	scienc	512	technolog	290
new	1903	world	502	random	290
influenza	1849	googl	442	infecti	277
relenza	1357	medic	422	home	265
pandem	1209	busi	418	viru	259
birdflu	851	symptom	409	daili	257
diseas	792	travel	385	children	250
life	789	music	373	care	250
vaccin	774	public	350	school	245
famili	739	person	349	govern	232
blog	739	obama	324	immun	230
cold	700	media	316	sport	223

**Table 2.** Degree and frequency of most frequent flu post bloggers, 5 October 2008 to 21 March 2009.

**Count**	**Blogger URL**	**Degree**		
		**In**	**Out**	**All**

**1,897**	crofsblogs.typepad.com/h5n1/	**64**	581	645
1,230	birdcauseflu.com	1	1	2
929	medblogs.org	0	6	6
912	afludiary.blogspot.com	30	659	689
359	healthinform3.livejournal.com	0	4	4
330	fluwikie2.com	35	839	874
204	birdflumonitor.com	0	**1,012**	1,012

**Table 3.** Closeness, betweenness and eigenvector centrality of the most frequent flu post bloggers, 5 October 2008 to 21 March 2009.

**Blogger URL**	**Centrality measures**				
	**Closeness**			**Betweenness**	**Eigenvector Pagerank**
	**In**	**Out**	**All**		

crofsblogs.typepad.com/h5n1	**0.0001103**	0.00100130	0.00055580	0.00003249	**0.00000373**
birdcauseflu.com	0.0000017	0.00000172	0.00000172	0.00000000	0.00000042
medblogs.org	0.0000000	0.00001034	0.00000517	0.00000000	0.00000042
afludiary.blogspot.com	0.0000517	0.00113572	0.00059371	**0.00005374**	0.00000149
healthinform3.livejournal.com	0.0000000	0.00000689	0.00000345	0.00000000	0.00000042
fluwikie2.com	0.0000603	0.00144593	0.00075313	0.00004468	0.00000042
birdflumonitor.com	0.0000000	**0.00174408**	0.00087204	0.00000000	0.00000042

**Table 4.** Six largest *influenza* content web and social media communities discovered by the Girvan-Newman community finding algorithm.

**Community Size**		**Closeness**			**Betweenness**	**Eigenvector Page Rank**
	**URL**	**IN**	**OUT**	**ALL**		
	**Personal blogs (google feeds and feedburner) & general-reporting newspapers**					
781	feeds.feedburner.com	0.4532	0.3802	0.2661	0.0533	**0.0347**
	www.google.org	0.4446	0.4098	0.2102	0.0127	0.0261
	www.nytimes.com	**0.5614**	**0.4954**	**0.3207**	**0.0931**	0.0116
	feedproxy.google.com	0.4751	0.3857	0.2852	0.0281	0.0089
	www.washingtonpost.com	0.5346	0.4749	0.3093	0.0321	0.0074
	**Mainstream network news, local news outlets**					
599	www.reuters.com	**0.5606**	**0.4887**	**0.3252**	**0.0767**	**0.0113**
	news.xinhuanet.com	0.5136	0.4589	0.3103	0.0292	0.0087
	online.wsj.com	0.5315	0.4766	0.3105	0.0306	0.0076
	www.bloomberg.com	0.5278	0.4676	0.3151	0.0310	0.0070
	www.foxnews.com	0.4952	0.4566	0.2894	0.0121	0.0061
	**Primary audience outside United States**					
397	news.bbc.co.uk	**0.5440**	**0.4855**	**0.3064**	0.0480	**0.0122**
	www.guardian.co.uk	0.5255	0.4683	0.3034	**0.0758**	0.0082
	www.telegraph.co.uk	0.5045	0.4571	0.2934	0.0159	0.0070
	news.google.co.uk	0.4629	0.4072	0.2887	0.0079	0.0052
	www.timesonline.co.uk	0.4906	0.4487	0.2855	0.0065	0.0051
	**Livejournal community and Entertainment industry (Viacom, Reed)**					
145	latimesblogs.latimes.com	0.4836	**0.4213**	0.2775	0.0046	**0.0025**
	community.livejournal.com	**0.4875**	0.2454	**0.3224**	**0.0415**	0.0017
	www.people.com	0.4170	0.3381	0.2542	0.0045	0.0010
	www.mtv.com	0.4491	0.3612	0.2784	0.0034	0.0006
	www.variety.com	0.3528	0.2954	0.2103	0.0006	0.0005
	**Large news conglomerates (News corp and Disney)**					
144	www.youtube.com	**0.5704**	**0.5463**	0.1737	**0.0096**	**0.0220**
	www.bloggingstocks.com	0.4023	0.3506	**0.2619**	0.0028	0.0005
	news.aol.com	0.3379	0.2731	0.2410	0.0034	0.0005
	sports.espn.go.com	0.4270	0.3558	0.2610	0.0024	0.0004
	www.dailyfinance.com	0.3996	0.2959	0.2652	0.0005	0.0004
	**Commentary, opinion, editorial**					
127	whatreallyhappened.com	0.4505	**0.4257**	0.2348	0.0374	**0.0159**
	www.prisonplanet.com	**0.4837**	0.3750	**0.3068**	**0.0622**	0.0142
	www.torontosun.com	0.4081	0.3617	0.2336	0.0001	0.0012
	www.legitgov.org	0.4287	0.3857	0.2540	0.0008	0.0010
	www.presstv.ir	0.4186	0.3813	0.1902	0.0004	0.0007

**Table 5.** Graph-based data mining using Subdue to detect structural anomalies that facilitate influenza-like-illness identification.

Week of:	Anomaly Found?	Unusual frequent substructures of publisher types, categories, or URLs.

5-Oct-2008		NA
12-Oct-2008		MySpace (URL), Mainstream Media (publisher type), Public Health (category)
19-Oct-2008		Mainstream Media (publisher type), Flickr (URL), prisonplanet (URL)
26-Oct-2008		MySpace (URL), Mainstream News (publisher type)
2-Nov-2008		MySpace (URL), Barack Obama (category)
9-Nov-2008		Mainstream Media (publisher type), Google FluTrends (URL)
16-Nov-2008		Mainstream Media (publisher type), Amazon (URL), Google FluTrends (URL)
23-Nov-2008		NA
30-Nov-2008		NA
7-Dec-2008	Yes	Yahoo Answers UK (URL), MySpace (URL), Fox News (URL)
14-Dec-2008		NA
21-Dec-2008		Fox News (URL), MySpace (URL), BirdFluMonitor (URL)
28-Dec-2008		N/A
4-Jan-2009	Yes	Strong presence of personal blog to blog substructures.
11-Jan-2009	Yes	Strong presence of personal blog to blog substructures.
18-Jan-2009		NA
25-Jan-2009	Yes	Forums (publisher type)
1-Feb-2009		NA
8-Feb-2009	Yes	Strong presence of personal blog to blog substructures.
15-Feb-2009	Yes	High presence of personal blog to blog substructures.
22-Feb-2009	Yes	High presence of personal blog to blog substructures.
1-Mar-2009	Yes	MySpace (URL) > 1500 substructures, Mainstream Media (publisher type)
8-Mar-2009	Yes	MySpace (URL) > 330 substructures, Mainstream Media (publisher type)
15-Mar-2009	Yes	Very high presence of personal blog to blog substructures.

**Table 6.** Novel Influenza H1N1/A Articles Posted per Week in 2009.

Week in 2009	17^th^	18^th^	19^th^	20^th^	21^st^	22^nd^	23^rd^	24^th^
	
# of Articles	5,591	108,038	61,341	26,256	19,224	37,938	14,393	27,502
